# Extent, Nature, and Risk Factors of Workplace Violence in Public Tertiary Hospitals in China: A Cross-Sectional Survey

**DOI:** 10.3390/ijerph120606801

**Published:** 2015-06-16

**Authors:** He Liu, Siqi Zhao, Mingli Jiao, Jingtao Wang, David H. Peters, Hong Qiao, Yuchong Zhao, Ye Li, Lei Song, Kai Xing, Yan Lu, Qunhong Wu

**Affiliations:** 1 Department of Health Policy and Hospital Management, School of Public Health, Harbin Medical University, 157 Baojian Road, Nangang District, Harbin 150081, China; E-Mails: lhhh314@126.com (H.L.); siqizhao1992@foxmail.com (S.Z.); Liye19832014@126.com (Y.L.); xkk507@126.com (K.X.); 2 Institute of Quantitative &Technical Economics, Chinese Academy of Social Science, 5 Jian Guo Men Nei Road, Dongcheng District, Beijing 100000, China; 3 Department of Epidemiology and Biostatistics, School of Public Health, Harbin Medical University, 157 Baojian Road, Nangang District, Harbin 150081, China; E-Mail: wangjingtao1990@163.com; 4 Department of International Health, Johns Hopkins Bloomberg School of Public Health, 615 N Wolfe St., Baltimore, MD 21205, USA; E-Mail: dpeters@jhu.edu; 5 Endocrine and Metabolic Diseases, The 2nd Affiliated Hospital of Harbin Medical University, 246 Xuefu Road, Nangang District, Harbin 150081, China; 6 Heilongjiang Nursing College, 209 Xuefu Road, Nangang District, Harbin 150081, China; E-Mail: zhaoyuchong@foxmail.com; 7 Department of Social Medicine, School of Public Health, Harbin Medical University, 157 Baojian Road, Nangang District, Harbin 150081, China; E-Mail: sll507@126.com; 8 School of Public Health, Jiamusi University, Jiamusi 154007, China; E-Mail: jmsly@163.com

**Keywords:** Chinese medicine hospitals, general hospitals, specialist hospitals, workplace violence, risk factor

## Abstract

Using a cross-sectional survey design from 11 public tertiary hospitals (a specialist hospital, four Chinese medicine hospitals, and six general hospitals) in the urban areas of Heilongjiang, we determined the nature of workplace violence that medical staff have encountered in Chinese hospitals and identified factors associated with those experiences of violence. A total of 1129 health workers participated. The specialist hospital had the highest prevalence of physical violence (35.4%), while the general hospitals had the highest prevalence of non-physical violence (76%). Inexperienced medical staff (*p* < 0.001) were more likely to suffer non-physical violence than physical violence in Chinese medicine hospitals compared to experienced staff. Medical units (*p* = 0.001) had a high risk of non-physical violence, while surgical units (*p* = 0.005) had a high risk of physical violence. In general hospitals, staff with higher levels of anxiety about workplace violence were more vulnerable to both physical violence (1.67, 95% CI 1.36–2.10) and non-physical violence (1.309, 95% CI 1.136–1.508) compared to those with lower levels of anxiety, while rotating shift workers had a higher odds of physical violence (2.2, 95% CI 1.21–4.17) and non-physical violence (1.65, 95% CI 1.13–2.41) compared to fixed day shift workers. Thus, prevention should focus not only on high-risk sections of hospitals, but also on the nature of the hospital itself.

## 1. Introduction

Workplace violence (WPV) has become an alarming phenomenon worldwide and one of the biggest public health concerns [1,2,3]. While there is no general agreement between researchers on its definition, workplace violence has been previously defined as “incidents where staff are abused, threatened or assaulted in circumstances related to their work, including commuting to and from work, involving an explicit or implicit challenge to their safety, well-being or health”. This definition was adopted from the joint study of the International Labour Office (ILO), International Council of Nurses (ICN), World Health Organisation (WHO), and Public Services International (PSI), which was conceived by the European Commission in Dublin in 1995 [1]. WPV crosses all boundaries, and it could happen to anybody. It can be divided into physical and non-physical types (with the latter comprising threats, sexual harassment, and verbal abuse) [4].

WPV studies mostly originate from mental health or emergency care settings. Research on emergency department and psychiatric settings in the US, Kuwait [1], and Taiwan [4] has revealed a high prevalence of violence in these departments [5]. The prevalence rates of physical violence (PV) in general hospitals in Australia and Brazil range from 3% to 17%. Meanwhile, for non-physical violence (NPV), the rates are as follows: for verbal abuse, 27.4%–67%; for bullying/mobbing, which is defined as repeated, unwanted, and unreasonable behaviour directed toward an employee, 10.5%–23%; for sexual harassment, 0.7%–8%; and for racial harassment, 0.8%–2.7% [6]. Research in England, Hong Kong, and China has shown that PV varied from 5.3% to 21% and verbal abuse from 43% to 73% [7].

### Background

In China, medical disputes are constantly emerging and appear to be worsening. Currently, WPV is occurring increasingly often in the Chinese medicine sector, with numerous reports of physicians being stabbed. This indicates that China is experiencing a unprecedented, nationwide crisis involving “patient safety”, “health care security”, and “physician–patient relations” [8].

The healthcare sector is at a significantly high risk for WPV [7]. The 2005 Chinese Hospital Association surveys reported that around 73.3% of hospital staff had been victims of such violence at some point or had been threatened or taunted by patients and their families [9]. In 2014, the national health institution clinics saw 7.8 billion patients, which was an increase of more than 0.5 billion in 2013. In that same year, the total number of national medical disputes was 115,000 (according to incomplete statistics), while the number of medical staff beaten or injured was more than 10,000 in China in 2014 [10]. The news media reported on the murders of 11 medical staff by patients between 2000 and 2010 [11]. However, the data actually reported are only the tip of the iceberg of hospital violence. As indicated by the rates of hospital medical injuries in the Workplace Violence Survey from the Chinese Hospital Association, the number of hospital violence incidents has been increasing [8]. 

Violent incidents result in serious adverse effects on the hospital, medical staff, and patients themselves, such as in a case in Wenling, China, wherein several physicians were killed. Violence and conflicts exist in every society; however, violence in medical settings not only results in harm to medical personnel but can also create potential crises for other patients. For instance, in the Shanghai Shuguang Hospital ICU, family members of a patient began smashing ICU medical emergency supplies and equipment and beat medical staff because of their displeasure with hospital treatment. Their violence caused numerous ill patients to become stressed by the attackers’ presence and several monitors to lose function. Without these monitors—connected to crucial life-sustaining technology—many ICU patients’ lives were put in danger [12]. As such, violence in medical establishments may lead to the deaths of patients not involved in the dispute. 

After physicians have been violently attacked, their mental status changes substantially; they may become unconcerned for patients, overprotective of their own lives, slacken in their professional roles, or overly negative [9]. Physicians might even organize formal protests to express their discontent. The most important impact of violence on medical staff is reflected in medical care strategies [8]: physicians who have been attacked will often begin to practice defensive medicine, wherein physicians avoid high-risk surgeries or patients to avoid risks to their own health and reduce the potential of another attack in the future. Regarding treatment choices, physicians begin to prioritize their own safety; one survey showed that even when facing critically ill patients who completely qualified for a given treatment, only 15% of physicians were willing to adhere to the “self-preservation first” strategy [10]. Often, this results in patients’ deaths. Furthermore, healthcare workers may be more conservative by not attempting more innovative initiatives, which can have an adverse impact on the diagnosis and treatment of complex diseases and critical illness. In the long run, this would have a devastating impact on the exploration and development of medical science [7,8,9].

WPV toward healthcare staff has been the subject of some research, although much of the existing evidence relates to psychiatric settings or mental healthcare units [13]. However, in recent years, WPV research in general hospitals [13] and other high-risk areas, such as elderly care institutions [14], has gradually increased. Most studies have focused on factors that predict patients’ violent behaviour (age, gender, presence of psychotic symptoms, personality traits, and previous drug abuse), while some researchers have looked at staff factors (professional, years of experience, contact with patients, anxiety level) to determine WPV risk factors [15].

However, few studies have considered specialist hospitals and Chinese medicine hospitals or have performed a horizontal comparison between the different types of hospitals. In China, hospitals can be classified by type: Chinese medicine hospitals (in which Chinese medicines are used as the main treatment modality), specialized hospitals (wherein the focus is on one or several sub-specialties, such as orthopaedics, psychiatry, dermatology, or obstetrics and gynaecology), and general hospitals (which treat a variety of diseases in numerous different departments; they should at least have emergency, internal medicine, surgical, obstetrics and gynaecology, and preventive healthcare departments).

Even the definition of WPV may differ between sociocultural environments, not to mention the manifestation of hospital violence and its causes, consequences, and intervention strategies [16]. The social climate and level of supportiveness in the workplace are also important determinants of recovery from work-related assaults. One study on the nature of medical institutions showed that the prevalence of WPV in public hospitals is higher than it is in private hospitals [17].

To date, almost no empirical studies have been published comparing different types of hospitals and the social, cultural, and organizational factors that might explain differences in WPV prevalence in Chinese public hospitals [18]. Vague reform policies in Chinese public hospitals have led to uneven distribution of medical resources, wherein high-quality resources are concentrated in public tertiary general hospitals. As such, many people, even those with minor illnesses, flock to large hospitals, which has resulted in unmanageable workloads for physicians, long waiting times, and short treatment times. Many physicians are struggling to cope, and this has ultimately affected the physician–patient relationship, which is a large cause of WPV [11]. In addition, due to low government compensation, hospitals must increase prescription costs and large equipment inspection to run their businesses, and because these costs are not covered by health insurance, patients have rather high out-of-pocket medical expenses. Indeed, research has shown that public tertiary general hospitals have the highest out-of-pocket medical expenses [13] and the poorest medical staff ethics, which have both increased the likelihood of WPV [9,15]. Higher-level hospitals have suffered from more serious WPV because these hospitals generally deal with more serious diseases, which in turn likely leads to higher stress levels among patients, family members, and staff. Our study was designed to identify the prevalence and risk factors of WPV against medical staff in public tertiary general, specialist, and Chinese medicine hospitals located in Heilongjiang Province, China.

## 2. Materials and Methods

### 2.1. Study Population

This study was conducted as a cross-sectional design in three districts in Heilongjiang Province, China. We purposively selected the cities of Harbin, Daqing, and Qiqihar for the sample based on the health statuses as well as the number of hospital beds and gross domestic products (GDPs) in 2014. All 64 public tertiary hospitals in these three regions were queried regarding their interest in participating in the survey. After hearing detailed explanations on the objective of this investigation, 11 hospitals expressed interest in participating in this survey. These 11 hospitals comprised different types of tertiary hospitals of the province: four Chinese medicine hospitals, six general hospitals, and one specialist orthopaedic hospital in the capital of Heilongjiang Province.

### 2.2. Sampling and Data Collection

Cooperation was obtained from managers in the tertiary hospitals and human resources departments. We selected 100 medical staff from the specialist hospital (response rate: 99%), 900 medical staff from the six general hospitals (response rate: 84.6%), and 320 medical staff from the four Chinese medicine hospitals (response rate: 83.4%). We used systematic random sampling to select the medical staff from a roster of all staff at each hospital. In the event of a person withdrawing, another was selected from the roster as a replacement. Data were collected from 15 July to 26 October 2014. A total of 1129 medical staff were selected from the 11 public tertiary hospitals, covering medical units, surgical units, and other departments (e.g., Ear, Nose, and Throat; administrative; medical technology) and a variety of shift types (e.g., rotating shift and fixed day shift). The questionnaire was self-administered.

### 2.3. Questionnaire

We used the questionnaire developed by the ILO, ICN, WHO, and PSI joint program in 2003 to measure WPV. First, we obtained permission from the ILO and WHO to use the questionnaire; then, we translated the questionnaire into Mandarin Chinese and invited 17 healthcare-related experts from all over China to evaluate its suitability for Chinese culture and the appropriateness of the translation. Two-week test-retest reliability was calculated (0.87) with a group of 37 healthcare workers in five hospitals. The questionnaire was then back translated into English in order to verify the accuracy of the Mandarin Chinese version. Data for analysis were collected with the final questionnaire, which has four sections:

1. Demographic and workplace data (gender, age in years, years of experience, professional, professional title, department), working in rotating shifts or not, and having contact with patients or their families in their daily work) and a question about anxiety about WPV, which was measured on a scale from 1 (not at all) to 5 (extremely high);
2. Questions about PV experiences (defined as use of physical force against another person or group that results in physical, sexual, or psychological harm) within the preceding year. This can include beating, kicking, slapping, stabbing, shooting, pushing, biting, or pinching.
3. Questions about NPV (defined as use of power, including threat of physical force, against another person or group that can result in harm to physical, mental, spiritual, moral, or social development) within the preceding year. Psychological violence includes verbal abuse, bullying/mobbing, harassment, and threats.
4. A multiple choice question to clarify respondents’ opinions on the reason for WPV: “What do you think causes WPV?” The reasons on the list included “not satisfied with high out-of-pocket medical expenses”, “physician–patient miscommunication”, “treatment is not satisfactory”, “patients do not meet all of the requirements for treatment”, “mistake by hospital medical staff”, “treatment time is too long/tedious treatment process” [9], “ulterior motives colleagues instigation”, “patient death”, “patients” condition suddenly deteriorates’, “physicians are overworked”, “information asymmetry between physicians and patients”, and “law enforcement officers did not take appropriate measures”.

### 2.4. Data Analysis

EpiData version 3.1 was used for data entry. Descriptive statistics were calculated to describe the demographic characteristics and frequency of the WPV reported. Relationships between staff background variables and prevalence of violence were detected using chi-square tests (χ^2^). Multiple logistic regression analysis was used to explore the association between the characteristics. Odds ratios (ORs) along with 95% confidence intervals were calculated as the measure of association between the outcomes and exposures. IBM SPSS Statistics 19.0 was used for analysis. Statistical significance was defined as *p* < 0.05.

### 2.5. Ethics Approval

The study protocol was reviewed and approved by the Research Ethics Committee of Harbin Medical University. Before the survey, approval was also obtained from each study hospital. All participants voluntarily and anonymously participated, and provided their written informed consent. To ensure anonymity, we prepared an envelope for each questionnaire and a box for collecting the questionnaires in the manager’s office of each hospital; furthermore, participants were never required to provide a name or other identifier. One of the authors was responsible for the collection box. The questionnaires were destroyed after data entry had been completed.

## 3. Results

### 3.1. Questionnaire Respondents

The demographic and professional characteristics of respondents are described in Table 1. Of the 1129 respondents, 64.7% (*n* = 731) were male. Additionally, 90.4% had experienced contact with patients or their families at work. Regarding age, 441 (39.1%) were between 26 and 35 years old. A total of 56.1% were physicians (*n* = 633), and 34.6% had a professional title of “intermediate” (*n* = 391). There were 446 respondents with fewer than 5 years of experience (39.5%), and 71.8% reported working rotating shifts. In the sample hospitals, medical staff undertaking shift work could be categorized into fixed day shift and rotating shift groups. Most nurses worked 8-h shifts; head nurses or nurse who had infants in the breastfeeding period worked fixed day shifts, although some hospitals extended this priority level for the latter group until children were 4 years of age. While, most physicians worked day shift and night shift, department director worked fixed day shift.About 39.2% reported an extremely high level of anxiety about WPV.

**Table 1:** Demographic characteristics of participants.

Characteristic	Chinese Medicine Hospitals (*n* = 269)		Specialist Hospitals (*n* = 99)		General Hospitals (*n* = 761)		Total (*n* = 1129)	
	*N*	%	*N*	%	*N*	%	*n*	%
**Gender**								
Male	73	27.1	47	47.5	278	36.5	398	35.3
Female	196	72.9	52	52.5	483	63.5	731	64.7
**Age groups (Years of Age)**								
≤25	62	23.0	16	16.2	311	40.9	389	34.5
26–35	108	40.1	39	39.4	294	38.6	441	39.1
>35	99	36.8	44	44.4	156	20.5	299	26.5
**Professional**								
Physicians	154	57.2	45	45.5	434	57.0	633	56.1
Nurses	85	31.6	37	37.4	267	35.1	389	34.5
Test technicians	8	3.0	14	14.1	25	3.3	47	4.2
Others	22	8.2	3	3.0	35	4.6	60	5.3
**Professional titles**								
Senior	10	3.7	5	5.1	88	11.6	103	9.1
Intermediate	69	25.7	28	28.3	294	38.6	391	34.6
junior	84	31.2	29	29.3	223	29.3	336	29.8
no	106	39.4	37	37.4	156	20.5	299	26.5
**Years of experience**								
≤5	95	35.3	12	12.1	339	44.5	446	39.5
6–20	84	31.2	35	35.4	263	34.6	382	33.8
≥21	90	33.5	52	52.5	159	20.9	301	26.7
**Working rotating shifts**								
Yes	169	62.8	81	81.8	561	73.7	811	71.8
No	100	37.2	18	18.2	200	26.3	318	28.2
**Department**								
Internal medicine	175	65.1	9	9.1	279	36.7	463	41.0
Surgical	36	13.4	68	68.7	297	39.0	401	35.5
Others	58	21.6	22	22.2	185	24.3	265	23.5
**Anxiety level**								
Extremely high	84	31.2	54	54.5	305	40.1	443	39.2
High	37	13.8	14	14.1	129	17.0	180	15.9
Moderate	93	34.6	18	18.2	230	30.2	341	30.2
Low	23	8.6	16	16.1	65	8.5	104	9.2
Not at all	32	11.9	7	7.1	32	4.2	71	6.3
**Contact with patients or their families in medical work**								
Yes	224	83.3	83	83.8	714	93.8	1021	90.4
No	45	16.7	16	16.2	47	6.2	108	9.6

### 3.2. Experience of WPV in the Preceding Year

Table 2 outlines the frequency of PV and NPV in each type of hospital. The highest prevalence of PV was in the specialist hospital (35.4%), followed by the general hospitals (14.1%) and the Chinese medicine hospitals (8.6%). The frequencies of NPV in the preceding year in the Chinese medicine hospitals, specialist hospital, and general hospitals were 76%, 75.8%, and 68%, respectively (Table 2).

**Table 2:** Frequency of WPV exposure.

	Chinese Medicine Hospitals (*n* = 269)				Specialist Hospitals (*n* = 99)				General Hospitals (*n* = 761)			
Exposure to violence	Physical Violence		Non-physical		Physical Violence		Non-physical		Physical Violence		Non-physical	
	*n*	%	*n*	%	*n*	%	*n*	%	*n*	%	*n*	%
Yes	23	8.6	183	68	35	35.4	75	75.8	107	14.1	578	76

### 3.3. Experiences of Aggression According to Occupational Characteristics

Table 3 shows the bivariate comparison of characteristics of medical staff who had experienced PV. Medical staff with an extremely high anxiety level about WPV (16.7%) and frequent contact with patients or their families (10.3%) were significantly more likely to have experienced PV in the Chinese medicine hospitals. 

In the specialist hospital, the only medical staff characteristic that predicted PV was the type of department. Specifically, participants from surgical units were significantly more likely to have been assaulted than were participants from other departments (χ^2^ = 10.435, *p* = 0.005).

In the general hospitals, medical staff had a higher likelihood of PV if they had years of experience between 6–20 (19.8%), were engaged in rotating shift work (16.6%), and had extremely high levels of anxiety about WPV (22.3%). Males were significantly more likely to be discharged from the general hospitals compared to females (17.6%).

**Table 3:** Characteristics and frequency distributions for physical violence (*N* = 1129).

Characteristic	Chinese Medicine Hospitals				Specialist Hospitals				General Hospitals			
	*n*	%	χ^2^	*p*	*n*	%	χ^2^	*p*	*n*	%	χ^2^	*p*
**Gender**												
Male	9	12.3	1.829	0.176	26	55.3	15.607	0.000	49	17.6	4.608	0.032
Female	14	7.1			9	17.3			58	12.0		
**Age groups (Years of Age)**												
≤25	8	12.9	3.123	0.210	2	12.5	10.749	0.005	39	12.5	4.514	0.105
26–35	10	9.3			21	53.8			51	17.3		
>35	5	5.1			12	27.3			17	10.9		
**Professional**												
Physicians	16	10.4	3.482	0.323	28	62.2	27.452	0.000	71	16.4	4.857	0.183
Nurses	7	8.2			3	8.1			31	11.6		
Test technicians	0	0.0			3	21.4			2	8.0		
Others	0	0.0			1	33.3			3	8.6		
**Professional titles**												
Senior	0	0.0	1.293	0.731	1	20.0	4.792	0.188	12	13.6	7.083	0.069
Intermediate	5	7.2			6	21.4			38	12.9		
Junior	8	9.5			11	37.9			42	18.8		
No	10	9.4			17	45.9			15	9.6		
**Years of experience**												
≤5	9	9.5	0.614	0.736	0	0.0	32.407	0.000	40	11.8	11.351	0.003
6–20	8	9.5			25	71.4			52	19.8		
≥21	6	6.7			10	19.2			15	9.4		
**Working rotating shifts**												
Yes	20	11.8	6.271	0.012	33	40.7	5.657	0.017	93	16.6	11.192	0.001
No	3	3.0			2	11.1			14	7.0		
**Department**												
Internal medicine	16	9.1	3.322	0.19	2	22.2	10.435	0.005	39	14.0	16.555	0.000
Surgical	5	13.9			31	45.6			57	19.2		
Others	2	3.4			2	9.1			11	5.9		
**Anxiety level**												
Extremely high	14	16.7	10.467	0.033	26	48.1	9.176	0.057	68	22.3	30.124	0.000
High	2	5.4			4	28.6			15	11.6		
Moderate	5	5.4			3	16.7			18	7.8		
Low	1	4.3			1	6.3			4	6.2		
Not at all	1	3.1			1	14.3			2	6.3		
**Contact with patients or their families in medical work**												
Yes	23	10.3	5.053	0.025	29	34.9	0.038	0.844	103	14.4	1.277	0.258
No	0	0.0			6	37.5			4	8.5		

The prevalence of NPV in Chinese medical hospitals was higher among participants engaged in rotating shift work (*p* < 0.001), which was the same for PV, and in participants with moderate levels of anxiety about WPV (*p* = 0.004), who were physicians (*p* = 0.002), who had less than 5 years of experience (*p* < 0.001), and who worked in an internal medicine department (*p* < 0.001) (Table 4).

In the specialist hospital, of the medical staff who worked in an internal medicine department (*n* = 8), 88.9% showed higher prevalence of NPV compared to PV. In the general hospital, medical staff with frequent contact with patients or their families (*p* = 0.002) had a higher prevalence of NPV than they did PV.

**Table 4:** Characteristics and frequency distributions for non-physical violence (*N* = 1129).

Characteristic	Chinese Medicine Hospitals				Specialist Hospitals				General Hospitals			
	*n*	%	χ^2^	*p*	*n*	%	χ^2^	*p*	*n*	%	χ^2^	*p*
**Gender**												
Male	55	75.3	2.463	1.117	41	87.2	6.417	0.011	215	77.3	0.460	0.497
Female	128	65.3			34	65.4			363	75.2		
**Age groups (Years of Age)**												
≤25	50	80.6	17.892	0.000	8	50.0	10.154	0.006	231	74.3	5.449	0.066
26–35	81	75.0			35	89.7			236	80.3		
>35	52	52.5			32	72.7			111	71.2		
**Professional**												
Physicians	112	72.7	15.014	0.002	42	93.3	14.059	0.003	333	76.7	5.444	0.142
Nurses	59	69.4			22	59.5			206	77.2		
Test technicians	5	62.5			9	64.3			18	72.0		
Others	7	31.8			2	66.7			21	60.0		
**Professional titles**												
Senior	7	70.0	6.119	0.106	2	40.0	4.679	0.197	67	76.1	0.753	0.861
Intermediate	50	72.5			20	71.4			223	75.9		
Junior	63	75.0			24	82.8			173	77.6		
No	63	59.4			29	78.4			115	73.7		
**Years of experience**												
≤5	74	77.9	17.972	0.000	6	50.0	7.668	0.022	249	73.5	4.057	0.132
6–20	63	75.0			31	88.6			211	80.2		
≥21	46	51.1			38	73.1			118	74.2		
**Working rotating shifts**												
Yes	132	78.1	21.224	0.000	63	77.8	0.990	0.320	445	79.3	13.273	0.000
No	51	51.0			12	66.7			133	66.5		
**Department**												
Internal medicine	133	76.0	15.336	0.000	8	88.9	14.254	0.001	210	75.3	0.618	0.734
Surgical	21	58.3			57	83.8			230	77.4		
Others	29	50.0			10	45.5			138	74.6		
**Anxiety level**												
Extremely high	63	75.0	15.445	0.004	41	75.9	6.665	0.155	246	80.7	22.932	0.000
High	24	64.9			13	92.9			105	81.4		
Moderate	70	75.3			14	77.8			169	73.5		
Low	10	43.5			4	25.0			42	64.6		
Not at all	16	50.0			3	42.9			16	50.0		
**Contact with patients or their families in medical work**												
Yes	164	73.2	16.549	0.000	64	77.1	0.510	0.475	551	77.2	9.393	0.002
No	19	42.2			11	68.8			27	57.4		

**Table 5:** Multiple logistic regression of physical and non-physical violence in Chinese hospitals, specialist hospital, general hospitals ^*^.

	Physical Violence									Non-Physical Violence								
	*Chinese Hospitals n* = 23			*specialist Hospital n* = 35			*general Hospitals n* =107			*Chinese Hospitals n* = 183			*specialist Hospital n = 75*			*general Hospital n*= 583		
	OR	95%CI	*p-*value	OR	95%CI	*p-*value	OR	95%CI	*p-*value	OR	95%CI	*p-*value	OR	95%CI	*p-*value	OR	95%CI	*p-*value
**Gender**	-	-	-	0.167	0.057–0.485	0.001	0.751	0.484–1.166	0.202	0.637	0.248–1.636	0.349	0.221	0.069–0.709	0.011	-	-	-
**Professional titles**	-	-	-	-	-	-	-	-	-	-	-	-	1.077	0.577–2.012	0.815	-	-	-
**Years of experience**	-	-	-	0.788	0.376–1.653	0.529	-	-	-	0.819	0.443–1.516	0.526	1.261	0.575–2.767	0.562	-	-	-
**Working rotating shifts**	3.648	1.043–12.766	0.043	3.687	0.688–19.760	0.128	2.161	1.183–3.947	0.012	3.434	0.902–13.073	0.071	-	-	-	1.647	1.127–2.406	0.010
**Department**	-	-	-	-	-	-	-	-	-	-	-	-	-	-	-	-	-	-
Surgical																		
Medicine				1.412	0.217–9.185	0.718	0.720	0.451–1.148	0.168	0.447	0.128–1.563	0.207	3.091	0.333–28.732	0.321	-	-	-
Others				0.144	0.029–0.725	0.019	0.312	0.155–0.627	0.001	0.251	0.043–1.482	0.127	0.174	0.056–0.542	0.003	-	-	-
**Anxiety level**	1.637	1.092–2.453	0.017	-	-	-	1.688	1.357–2.100	<0.001	1.665	1.095–2.533	0.017	-	-	-	1.309	1.136–1.508	<0.001
**Contact with patients or their families in medical work**	-	-	-	-	-	-	-	-	-	-	-	-	-	-	-	1.745	0.914–3.333	0.092

* This analysis used data from 1129 medical staff from 11 tertiary hospitals in Heilongjiang; - Represents the variables have not been put in the model because they are not significant in the Chi-square text.

### 3.4. Multiple Logistic Regression Analysis Predicting WPV

In view of the results shown in Table 2, we hypothesized that different types of hospitals have different risk factors. Thus, we put the significant results of the chi-square test (Table 3 and Table 4) into logistic regression models. Table 5 shows the results of the multiple logistic regression analyses for PV and NPV (Table 5).

The odds of experiencing PV (0.17, 95% CI 0.057-0.485) and NPV (0.22, 95% CI 0.069–0.709, *p* = 0.011) were higher for men in the specialist hospital. Rotating shift workers had an over three-fold greater odds of PV (3.65, 95% CI 1.04–12.76) compared to fixed day shift workers in the Chinese medicine hospitals. Furthermore, in the general hospitals, rotating shift workers had an over two-fold greater odds of experiencing PV (2.16, 95% CI 1.18–3.95) and an over 1.5-fold greater odds of experiencing NPV (1.65, 95% CI 1.13–2.4) compared to fixed day shift workers. 

Furthermore, medical staff in the Chinese medicine hospitals who were highly anxious about WPV had an over 1.5-fold greater odds of experiencing both PV (1.64, 95% CI 1.09–2.45) and NPV (1.66, 95% CI 1.1–2.53).

### 3.5. Reasons for WPV

Most medical staff in Chinese hospitals who had experienced WPV reported that the main reason for the violence had been “patients did not meet all the requirements for treatment” (*p* = 0.036). In contrast, the staff of the specialist hospital viewed such violence as being due to “high out-of-pocket medical expenses” (*p* = 0.02), while in general hospitals it was because “physicians were overworked” (*p* < 0.001) (Table 6). 

**Table 6:** Reasons for WPV according to medical staff who had experienced WPV.

	Reasons	n	%	χ^2^	*p*
**CHi**	**Patients did not meet all the requirements for treatment**	16	69.6	4.383	0.036
**SPe**	**not satisfactory with high out-of-pocket medical expenses**	13	37.1	5.849	0.016
	**Cognitive gap between physicians and patients**	27	57.1	4.874	0.027
	**Law enforcement officers did not take coercive measures**	20	37.1	9.040	0.003
**GEn**	**physicians labor-intensive**	49	73.8	21.737	0.000
	Law enforcement officers did not take coercive measures	50	62.6	3.924	0.048
	Patients did not meet all the requirements	62	57.9	9.604	0.002
	**Waiting time too long**	52	48.6	5.215	0.022
	Cognitive gap between physicians and patients	79	46.7	10.759	0.001

## 4. Discussion

An analysis of the responses from the 1129 medical staff from the Chinese medicine hospitals, specialist hospital, and general hospitals regarding their experience of WPV in the preceding year found that the prevalence differed by factors such as age, profession types, departments, and gender. Thus, the prevalence of WPV was high overall [17].

The most important finding identified in this analysis was that the prevalence of WPV differed by hospital type. The highest prevalence of PV in the preceding year occurred in the specialist hospital (*n* = 35, 35.4%), compared with the general hospitals (*n* = 107, 14.1%) and Chinese medicine hospitals (*n* = 23; 8.6%). In contrast, the prevalence of NPV in the preceding year was as follows: general hospitals (*n* = 578, 76%), specialist hospital (*n* = 75, 75.8%), and Chinese medicine hospitals (*n* = 183, 68%). The significance of our study is that, in addition to previous studies of WPV in the high-risk hospital departments (e.g., the emergency department, ICUs, and psychiatry [10,16]), the surgical departments (general hospital) and bone surgery departments (specialist hospital) may also be targets for intervention. 

In Chinese medicine hospitals, the results may reflect an actually general level prevalence of aggression, but an increased awareness of WPV might also have influenced recall [11]; as such, hospitals’ atmospheres and medical philosophies and patients’ perceptions of such hospitals all might have determined the reported prevalence of WPV. Traditional Chinese medicine espouses certain values and is highly humanistic; specifically, physicians must be dedicated to treatment and without desire or expectations regarding the same; they must also be compassionate and determined to decrease human suffering, and cannot consider their own gains and losses [7]. People who visit a physician in a Chinese medicine hospital generally have a “chronic illness”, and are already prepared that the disease will have a long recovery period. 

The development of technology and modernization of medical equipment and the increasing amount of media has led to excessive use of large medical equipment, which in turn has caused physicians to ignore patients’ feelings, thereby straining the physician–patient relationship [15]. Compared with treatment concepts from Western medicine, Chinese medicine treatments take a holistic view of the patient. Chinese medicine physicians are expected to give patients the opportunity to describe their illness, which allows physicians to understand the illness, make a diagnosis, and provide a holistic treatment [19]. Another reason that the prevalence of WPV in the Chinese medicine hospitals was lower compared to the general hospitals might be that the number of outpatients and the bed turnover rate of Chinese medicine hospitals are lower than in general hospitals; this leads to medical staff having more prolonged contact with patients and their families. With medical technology rapidly developing in Western medicine, especially in tertiary hospitals, poor communication between medical providers (including staff) and patients and distrust of physicians has been worsening [20]. 

The importance of the major factors predicting WPV prevalence, such as gender, profession type, years of experience, shift-work status, anxiety level, and contact with patients [21], becomes more apparent when the departments and professions are looked at separately. It is interesting that the risk factors differ between the hospital types. The ILO has noted that young people who lack work experience are more likely to become victims of WPV [21]. Coinciding with these previous studies, we found that inexperienced medical staff (77.9%, *p <* 0.001) were more likely to experience NPV than they were to experience PV in Chinese hospitals. This may be because perpetrators are reluctant to physically assault young medical staff, particularly female staff; in contrast, patients may feel more able to physically assault older staff who are more influenced by traditional ideas involving arbitrary medical treatment, which may result in high dissatisfaction in patients [9].

Our study has found that surgical units are a high-risk sector along with mental health and emergency care units. However, it also depends on the type of hospital and WPV. Internal medicine units (88.9%, *p* = 0.001) were high-risk places for NPV compared with surgical units in general hospitals, while the surgical unit (45.6%, *p* = 0.005) exhibited more PV compared with internal medicine units in the specialist hospital. Even so, it seems unlikely that the hospital type alone could account for the levels of aggression. This emphasizes the need for more detailed assessments of incidents to determine exactly what might be different in those hospitals or professions with high prevalence rates.

Contrary to previous studies [22], physicians, of all professions examined in this study, were most at risk from aggression in the Chinese hospitals and the specialist hospital compared with the general hospitals. The reason for this difference by hospital type might be that physicians in the Chinese medicine hospitals and specialist hospital have prolonged contact with patients [23]. 

Our multivariate logistic results indicated that gender, shift-work status, departments, and anxiety levels influenced the risk of exposure to WPV. Our study also found that male staff had the highest risk of exposure to PV, which is consistent with findings in other countries. Contrary to expectations [12], male staff were particularly vulnerable to NPV in the specialist hospital. Rotating shift workers had an over three-fold greater odds of PV compared to fixed day shift workers in the Chinese medicine hospitals, and an over two-fold greater odds of PV and an over 1.5-fold odds of experiencing NPV compared to fixed day shift workers in general hospitals. These findings concurred with previous studies that noted that working night shifts was considered a high-risk factor predicting exposure to violence [24].

The research suggests that prior experience of may influence how one responds to aggression in the future [25]. One of the most important reasons for WPV according to medical staff who had experienced it was “patients did not meet all the requirements for treatment” (69.6%). Some patients in China usually hold the opinion that since they have paid for medical services out of pocket, they must achieve good health outcomes. If the diagnosis and treatment do not achieve their intended effect, patients may feel that the hospital should not shirk its responsibility [26].

The specialist hospital victims held the opinion that PV occurs because of “not satisfactory with high out-of-pocket medical expenses” (37.1%), “cognitive gap between physicians and patients” (57.1%), and “law enforcement officers did not take coercive measures” (37.1%). Most hospitals in China, especially the large ones (e.g., Peking Union Medical College Hospital) are run by the government, and after recent economic reforms, many hospitals receive very limited financial support from the government [9]. This has resulted in hospitals, especially Chinese medicine hospitals and specialist hospitals, needing to generate income to cover costs. Low economic status may also have promoted WPV prevalence [21]. At present, low economic statuses and higher healthcare costs have put considerable financial pressure on patients. In addition, the high rate of Medicare co-payments has increased the burden on patients and their families. Patients want the best medicine and the best treatment, but because of the lack of economic resources, most tend to experience frustration and irritability, and often complain that the costs are too high.

Those who experienced WPV in general hospitals noted that the following factors might contribute to WPV: “physicians were overworked” (73.8%), “patients did not meet all the requirements for treatment” (57.9%), and “waiting time was too long” (48.6%). Traditionally, Chinese people have tended to seek high-quality medical care, even if they have not had a serious illness [19]. Patients often rush to secondary and tertiary hospitals with unrealistic expectations [9], making tertiary hospitals overcrowded and vastly increasing the workloads of medical staff in such hospitals; as such, many staff have only a few minutes with each patient, which is sufficient for appropriate communication. Without communication, patients have insufficient knowledge of the diseases, costs, and effects of treatment, which may result in their misunderstanding the treatment and ultimately to violence.

One other factor that might play a contributory role in WPV against health workers is “bad publicity”, which might cause people to view medical institutions as unsatisfactory [25]. Evidence from other studies has supported this argument [27]. Currently, the media have exaggerated negative medical and health events, health system failures, and bribe-taking cases; some have even gone so far as to place responsibility on all medical workers, which often leads to patients’ dissatisfaction with the entire medical institution. This could, then, cause WPV. There are, however, deeper causes of the hospital violence in China, including a lack of trust within society, gaps in the supply and demand of high-quality medical resources, and the social health insurance system [28]. 

A limitation of the present study was that the data were sampled from only one area of China. Thus, the results may, to some extent, reflect contextual factors not shared by other regions. Despite our strict survey quality control, there is inevitable error in the prevalence rates of WPV, as participants answered according to their perceptions. In addition, there still exist the risk for Type I error due to the large number of statistical tests that were performed. Nevertheless, this study provided initial evidence on the nature of WPV in China.

## 5. Implications for Practice

Much research on WPV in healthcare workers has focused on one type of hospital. However, this study demonstrated that different types of hospitals and units have different prevalence rates of WPV. Thus, prevention should focus not only on high-risk units in hospitals, but also on the type of hospital itself. Surgical (general hospital) and bone surgery (specialist hospital) departments may be important targets for PV interventions. We thus recommend that hospital management consider providing suitable education programs for high-risk groups about the expected risk of WPV and implement follow-up plans to support employees and prevent future occurrences.

## References

[1] Shen H.C., Cheng Y., Tsai P.J., Lee S.H., Guo Y.L. (2005). Occupational stress in nurses in psychiatric institutions in Taiwan. J. Occup. Health.

[2] Finnis S.J., Robbins I. (1994). Sexual harassment of nurses: An occupational hazard?. J. Clin. Nurs..

[3] Findorff M.J., McGovern P.M., Wall M., Gerberich S.G., Alexander B. (2004). Risk factors for work related violence in a health care organization. Inj. Prev..

[4] Lin Y.H., Liu H.E. (2005). The impact of workplace violence on nurses in South Taiwan. Int. J. Nurs. Stud..

[5] Cox H. (1991). Verbal abuse nationwide, Part II: Impact and modifications. Nurs. Manag..

[6] Atawneh F.A., Zahid M.A., al-Sahlawi K.S., Shahid A.A., Al-Farrah M.H. (2003). Violence against nurses in hospitals: Prevalence and effects. Brit. J. Nurs..

[7] Wenzhi C., Ling D., Meng L., Min Y. (2011). Antecedents of medical workplace violence in South China. J. Interpers. Violence.

[8] Zhou P., Zeng F., Liu S. (2014). “Injured” Chinese otolaryngologist. Eur. Arch. Otorhinolaryngol..

[9] Chen Z.H., Wang S.Y., Jing C.X. (2003). Prevalence of workplace violence in staff of two hospitals in Guangzhou. Zhonghua Yu Fang Yi Xue Za Zhi.

[10] Hegney D., Tuckett A., Parker D., Eley R.M. (2010). Workplace violence: Differences in perceptions of nursing work between those exposed and those not exposed: A cross-sector analysis. Int. J. Nurs. Pract..

[11] Yang L.Q., Spector P.E., Chang C.H., Gallant-Roman M., Powell J. (2012). Psychosocial precursors and physical consequences of workplace violence towards nurses: A longitudinal examination with naturally occurring groups in hospital settings. Int. J. Nurs. Stud..

[12] Danesh V.C., Malvey D., Fottler M.D. (2008). Hidden workplace violence: What your nurses may not be telling you. Health Care Manag. (Frederick).

[13] Hahn S., Muller M., Hantikainen V., Kok G., Dassen T., Halfens R.J. (2013). Risk factors associated with patient and visitor violence in general hospitals: Results of a multiple regression analysis. Int. J. Nurs. Stud..

[14] Astrom S., Bucht G., Eisemann M., Norberg A., Saveman B.I. (2002). Incidence of violence towards staff caring for the elderly. Scand. J. Caring Sci..

[15] Mingli J., Ning N., Ye L., Lijun G., Yu C., Hong S., Zheng K., Libo L., Qunhong W., Yanhua H. (2015). Workplace violence against nurses in Chinese hospitals: A cross-sectional survey. BMJ.

[16] Kling R.N., Yassi A., Smailes E., Lovato C.Y., Koehoorn M. (2011). Evaluation of a violence risk assessment system (the Alert System) for reducing violence in an acute hospital: A before and after study. Int. J. Nurs. Stud..

[17] Laschinger H.K., Grau A.L. (2012). The influence of personal dispositional factors and organizational resources on workplace violence, burnout, and health outcomes in new graduate nurses: A cross-sectional study. Int. J. Nurs. Stud..

[18] U.S. Department of Labor Guidelines for preventing workplace violence for health care and social service workers. https://www.osha.gov/newsrelease/nat-20150403.html.

[19] Hesketh T., Wu D., Mao L., Ma N. (2012). Violence against doctors in China. BMJ.

[20] Chen W.C., Hwu H.G., Kung S.M., Chiu H.J., Wang J.D. (2008). Prevalence and determinants of workplace violence of health care workers in a psychiatric hospital in Taiwan. J. Occup. Health.

[21] Winstanley S., Whittington R. (2002). Violence in a general hospital: Comparison of assailant and other assault-related factors on accident and emergency and inpatient wards. Acta Psychiatr. Scand. Suppl..

[22] Carmi-Iluz T., Peleg R., Freud T., Shvartzman P. (2005). Verbal and physical violence towards hospital- and community-based physicians in the Negev: An observational study. BMC Health Serv. Res..

[23] Hewitt J.B., Levin P.F. (1997). Violence in the workplace. Annu. Rev. Nurs. Res..

[24] Kitaneh M., Hamdan M. (2012). Workplace violence against physicians and nurses in Palestinian public hospitals: A cross-sectional study. BMC Health Serv. Res..

[25] Li Y., Wu Q., Xu L., Legge D., Hao Y., Gao L., Ning N., Wan G. (2012). Factors affecting catastrophic health expenditure and impoverishment from medical expenses in China: Policy implications of universal health insurance. Bull. WHO.

[26] The Lancet (2012). Ending violence against doctors in China. Lancet.

[27] Large M.M., Ryan C.J. (2015). Violence risk assessment has not been shown to reduce violence. Aust. N. Z. J. Psychiat..

[28] Roche M., Duffield C., White E. (2011). Factors in the practice environment of nurses working in inpatient mental health: A partial least squares path modeling approach. Int. J. Nurs. Stud..

